# Anesthetic management and outcomes in patients undergoing epicardial ablation for the treatment of ventricular tachycardia: A retrospective analysis

**DOI:** 10.1097/MD.0000000000045961

**Published:** 2025-11-14

**Authors:** Ferda Yaman, Tuba Akyüz, Ezgi Çamli Babayiğit, Taner Ulus

**Affiliations:** a Department of Anesthesiology and Reanimation, Faculty of Medicine, University of Eskisehir Osmangazi, Eskisehir, Turkey; b Cardiology Department, Eskisehir City Hospital, Eskisehir, Turkey; c Department of Cardiology, Faculty of Medicine, University of Eskisehir Osmangazi, Eskisehir, Turkey.

**Keywords:** catheter ablation, electro-anatomic mapping, electrophysiology, general anesthesia, risk assessment, ventricular tachycardia

## Abstract

Ventricular arrhythmias are an important cause of morbidity and mortality, manifesting in various forms, ranging from single premature ventricular complexes to sustained ventricular tachycardia and fibrillation. General anesthesia provides advantages in terms of secured airway, increased patient comfort and satisfaction, and controlled respiration; however, its disadvantages are exacerbated by compromised cardiopulmonary function, typically requiring vasopressors and inotropic support. This study aimed to examine the anesthetic challenges and risk assessment in patients receiving epicardial ablation for ventricular tachycardia. In this retrospective, observational study, we collected data by examining the files of all patients who underwent percutaneous epicardial catheter ablation in our electrophysiology unit between October 30, 2020, and October 30, 2022, after obtaining ethics committee approval. All procedures were performed under general anesthesia. Midazolam, fentanyl, and low-dose propofol were used for induction. Maintenance was achieved using sevoflurane. The baseline characteristics of the patients and follow-up periods were recorded. Twenty patients were eligible for inclusion in the study. The mean age of the patients was 63.0 (57.5–66.0) years, 17 patients were male, and the mean body mass index was 25.9 (25.0–27.5) kg/m^2^. The mean American Society of Anesthesiologists physical status classification score was 4. Fourteen cases were evaluated as New York Heart Association classes III-IV. The mean left ventricular ejection fraction was 22.5% (16.5–43.0). Seventeen patients required dopamine, and cardiopulmonary resuscitation was performed in 1 patient. No procedure-related deaths were observed in any of the patients. Anesthesiologists and cardiologists should take a personalized approach to patient care, beginning with the preoperative process and continuing through the post-procedure period, ensuring good communication and collaborative planning.

## 1. Introduction

Despite ongoing research and development of new therapeutic approaches, ventricular arrhythmias remain a major contributor to cardiac morbidity and mortality worldwide. Catheter ablation has played an increasingly prominent role in the treatment of ventricular tachycardia (VT).^[1]^ Subxiphoid percutaneous epicardial mapping and/or ablation, first described by Sosa et al in 1996, has developed into a significant adjunct, and in certain situations, the recommended treatment approach for several cardiac dysrhythmias, such as scar-mediated VT, accessory pathways, and idiopathic VT.^[2–4]^

Modern management of patients with ventricular arrhythmias requires a multidisciplinary team approach, especially in cases involving complex presentations and multiple medical comorbidities.^[5]^

Radiofrequency catheter ablation combined with electroanatomic mapping is increasingly being used to treat VT. Anesthesiologists are frequently required to manage the perioperative care of these patients in electrophysiology (EP) laboratories.^[6]^ Anesthetic management of epicardial VT ablation presents several challenges, as these patients often have ventricular dysfunction and are prone to excessive hypotension with anesthetic agents. In addition, epicardial access can be painful, and patient movement may interfere with electroanatomic mapping, resulting in recording errors.^[7,8]^ This retrospective, observational study involved the evaluation of the risk profile of patients with life-threatening VT in the EP and identification of key patient characteristics to emphasize the importance of anesthetic management through cardiac risk assessment during epicardial ablation under general anesthesia (GA) and to ascertain complications that developed during the procedure.

We aimed to define the techniques of the anesthetic approach before, during, and after the operation, and how they affect the process results in patients undergoing subxiphoid epicardial catheter ablation due to sustained VT. In addition, we aimed to present the follow-up data after subxiphoid epicardial ablation for VT.

## 2. Materials and methods

In this retrospective, observational study, data were collected by examining the files of all patients who underwent epicardial ablation for sustained VT under GA in the EP laboratory from October 30, 2020, to October 30, 2022, after obtaining approval from the ethics committee of Eskişehir Osmangazi University (approval number: 2022/33) (Trial registration: NCT06538987/2024-08-06). Obtaining informed consent from all study participants was challenging because this observational, retrospective study was conducted using medical records. The need for informed consent was waived by the Ethics Committee of Eskişehir Osmangazi University. All procedures in this study were performed in accordance with relevant guidelines and regulations. The baseline characteristics of the patients, including sex, age, body mass index (kg/m^2^), presence of comorbidities (hypertension, coronary artery disease, chronic obstructive pulmonary disease, and chronic kidney disease), electrical storms, and VT etiology (ischemic cardiomyopathy, dilated cardiomyopathy, arrhythmogenic right ventricular (RV) cardiomyopathy, and hypertrophic cardiomyopathy), were recorded. Cardiac magnetic resonance imaging (MRI) findings of the patients were recorded, if available.

Patients with serious comorbidities that limited their quality of life, previous epicardial ablation, lost to follow-up, or missing follow-up data were excluded from the study. Emergency patients with electrical storms were included in the study. Patients were categorized into 2 groups according to the New York Heart Association (NHYA): NHYA I/II and NHYA III/IV. Furthermore, the estimated glomerular filtration rate, left ventricular (LV) ejection fraction (LVEF), risk of acute hemodynamic decompensation (PAINESD), and I-VT score (risk of VT recurrence) were recorded. VT focus locations; the need for dopamine, dobutamine, and norepinephrine infusions during the procedure; the development of arrest during the procedure; and fluoroscopy and ablation times were also noted. Risk stratification was evaluated through the PAINESD and I-VT scores. The PAINESD score is a composite tool designed to predict periprocedural acute haemodynamic decompensation during VT ablation. The I-VT (Inducibility-VT) score is intended to assess the long-term risk of recurrence and outcomes following VT ablation. In contrast to PAINESD, which prioritizes acute intraprocedural haemodynamic risk, the I-VT score highlights the significance of the electrophysiological substrate and arrhythmia burden in assessing prognosis and procedural success. The scores were calculated and analyzed independently to differentiate between predictors of short-term intraprocedural instability and long-term arrhythmic outcomes.^[9]^

An epicardial procedure via the anterior route was conducted. A “dry” subxiphoid puncture was executed under fluoroscopic guidance in both anteroposterior and left anterior oblique 90° views. Following the first puncture of the skin beneath the xiphoid process, the needle was subsequently navigated in a medioclavicular trajectory towards the pericardial area under left anterior oblique 90° guidance to delineate the triangle formed by the sternum, abdominal organs, and right ventricle. The needle was gradually inserted directly beneath the sternum, yet above the abdominal organs. Verification of the accurate needle placement was conducted using contrast medium, and a lengthy guidewire was progressed into the epicardial area. Furthermore, dilation of the puncture site and the insertion of a long 8.5F SL-1 or a steerable sheath constituted the concluding procedures.^[10,11]^

The 3-dimensional mapping systems CARTO^TM^ (Biosense Webster) and ENSITE Precision^TM^ (Abbott) were utilized throughout the processes. Briefly, the cardiology team performed the previously specified VT ablation operations. Initially, they defined possible regions responsible for VT using voltage mapping, late-potential mapping, isochronal late-potential activation mapping, and decrement-evoked potential mapping. They then attempted to create clinical VT and performed activation mapping when VT occurred. Then, using substrate mapping and activation mapping, they performed ablation. Ablation based only on substrate mapping was performed if the patient did not tolerate VT or if clinical VT was not induced. In the post-procedural follow-up, the causes of death, development of decompensated heart failure, transient ischemic attacks, acute renal failure, and recurrence of VT were tracked. PAINESD score (acute hemodynamic decompensation risk assessment score (PAINESD) was recorded. The PAINESD score, developed to estimate the risk of periprocedural hemodynamic decompensation, ranges from 0 to 35 points (or 0 to 31 points [PAINESD] when the modifiable intraprocedural variable GA is excluded). Additional data recorded included the procedure duration, inotrope requirement during the procedure, mechanical ventilation requirement after the procedure, and procedural success rate.

The anesthetic approach applied before and during the procedure was tailored to each patient. Midazolam, propofol, fentanyl, and rocuronium were used to induce GA, and maintenance was achieved using sevoflurane. In the EP laboratory, anesthesia induction in all patients was achieved with minimal doses of propofol after the administration of 0.05 to 0.15 mg/kg midazolam and 1 mcg/kg fentanyl to reduce the propofol dose.

The patients were protected from hypothermia by heating their blankets. End-tidal carbon dioxide, tidal volume, urine output, and arterial blood gas were monitored through invasive arterial monitoring in accordance with the American Society of Anesthesiologists standards. All patients except 2 were extubated in the EP laboratory and transferred to the intensive care unit. VT recurrence was evaluated and documented during routine device interrogations in 16 patients who already had an ICD. In patients without an ICD, arrhythmic events were assessed using 24-hour Holter monitoring.

## 3. Statistical analysis

Descriptive statistics, including mean, standard deviation, median, minimum, maximum, frequency, and ratios, were used to analyze the data. SPSS version 28.0 (Chicago) was utilized for statistical analysis.

## 4. Results

**Figure 1.**
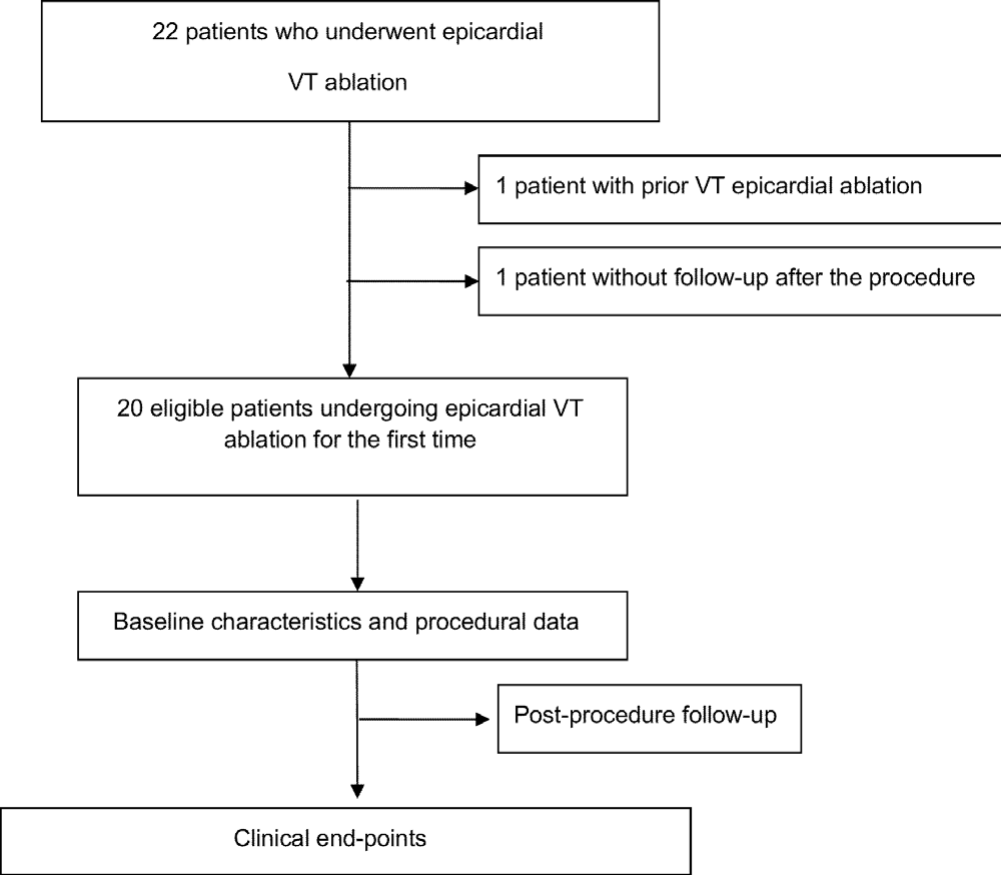
Flow-chart of the study. VT = ventricular tachycardia.

**Figure 2.**
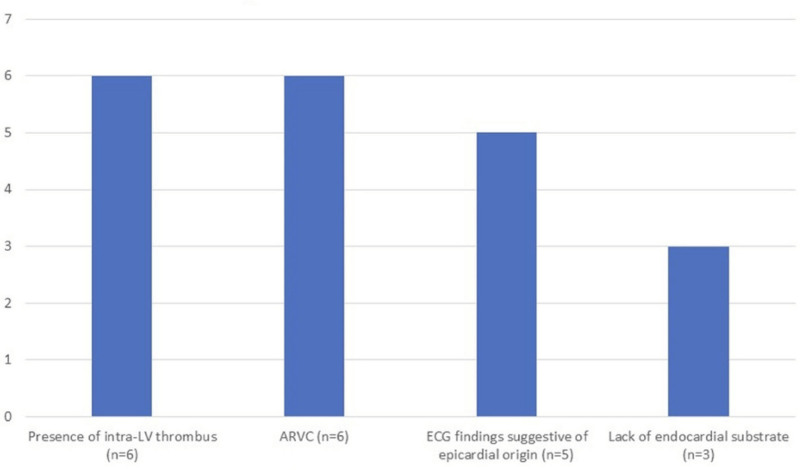
Indications for epicardial intervention in patients undergoing VT ablation. ARVC = arrhythmogenic right ventricular cardiomyopathy, ECG = electrocardiography, LV = left ventricle, VT = ventricular tachycardia.

A total of 22 patients were admitted to the electrophysiology laboratory, with 20 meeting the eligibility criteria. Figure 1 presents the flowchart of the study. Data were analyzed across 3 categories: pre-, intra-, and post-procedure. Among patients undergoing VT ablation, indications for epicardial intervention included arrhythmogenic RV cardiomyopathy, lack of endocardial substrate, presence of LV thrombus, and electrocardiography findings suggesting an epicardial origin (Fig. 2).

According to the pre-procedural evaluation, the mean age of the patients was 63.0 (57.5–66.0) years, 17 patients were male, and the mean body mass index was 25.9 (25.0–27.5) kg/m^2^. The mean American Society of Anesthesiologists physical status classification score was 4, and 14 cases were classified as NYHA class III to IV. The mean (LVEF) was 22.5% (16.5–43.0). The mean (PAINESD) score was 16.2 ± 9.3. Sixteen patients had implantable cardioverter-defibrillators (Table 1). The I-VT score for VT recurrence risk was 1.3 (0.6–1.3), while the I-VT score for VT death risk was 1.7 (0.1–2.8). VT recurrence during follow-up occurred in 5 (25.0%) patients. Cardiac MRI was performed in 8 patients before epicardial ablation. Of these, the etiology was arrhythmogenic cardiomyopathy in 4 patients, ischemic cardiomyopathy in 2 patients, hypertrophic cardiomyopathy in 1 patient, and idiopathic VT in 1 patient. Among the patients with arrhythmogenic cardiomyopathy, 2 had RV free wall sub-epicardial late Gadolinium enhancement (LGE), one had RV free wall sub-epicardial and LV mid-myocardial LGE, and one had RV mid-myocardial and LV transmural LGE. LV subendocardial LGE was observed in both patients with ischemic cardiomyopathy. Apical transmural LGE was detected in the patient with hypertrophic cardiomyopathy. Cardiac MRI could not be performed in the other patients because they had a preexisting ICD and the system was not MRI compatible.

**Table 1:** Baseline characteristics of the patients (n = 20).

Sex (male) (n,%)	17 (85.0)
Age (year)	63.0 (57.5–66.0) 58.4 ± 13.6
BMI (kg/m^2^)	25.9 (25.0–27.5) 28.0 ± 5.9
Hypertension (n,%)	14 (70.0)
Diabetes mellitus (n,%)	5 (25.0)
Coronary artery disease (n,%)	7 (35.0)
COPD (n,%)	4 (16.0)
CKD (n,%)	5 (25.0)
Electrical storm (n,%)	10 (50.0)
Etiology	
Ischemic cardiomyopathy (n,%)	8 (40.0)
Dilated cardiomyopathy (n,%)	5 (25.0)
ARVC (n,%)	6 (30.0)
Hypertrophic cardiomyopathy (n,%)	1 (5.0)
Aneurysm	11 (55.0)
LV Apical aneurysm (n,%)	7 (35.0)
RV aneurysm (n,%)	4 (20.0)
LV thrombus (n,%)	6 (30.0)
NHYA class	
NHYA I or II	6 (30.0)
NHYA III or IV	14 (70.0)
ICD (n,%)	16 (80.0)
CRT-D (n,%)	4 (20.0)
Hemoglobin (g/dl)	13.5 ± 1.7
eGFR	81.8 (57.5–90.0) 74.1 ± 16.9
LV EF (%)	22.5 (16.5–43.0) 32.2 ± 17.5
LA diameter (mm)	41.0 (35.0–47.5) 43.1 ± 9.7
Systolic pulmonary artery pressure (mm Hg)	28.5 (25.0–42.0) 33 ± 10.3
PAAINESD Score	16.2 ± 9.3
I-VT score (risk of VT recurrence)	1.3 (0.6–1.3)
I-VT score (risk of VT death)	1.7 (0.1–2.8)
ASA Score	4.0 (3.0–4.0)

Categorical variables are presented as number (percentage), and continuous variables are expressed as mean ± standard deviation. (min–max, median, mean ± SD, n,%).

ARVC = arrhythmogenic right ventricular cardiomyopathy, BMI = body mass index, CKD = chronic kidney disease, COPD = chronic obstructive pulmonary disease, CRT-D = cardiac resynchronization therapy with defibrillator function, EF = ejection fraction, eGFR = estimated glomerular filtration rate, ICD = implantable cardioverter defibrillator, LA = left atrium, LV = left ventricle, NYHA = New York Heart Association, RV = right ventricle, SD = standard deviation, VT = ventricular tachycardia.

During the procedure, 1 patient developed cardiopulmonary arrest, and 17 patients required dopamine, dobutamine, and norepinephrine infusions to maintain hemodynamics. No procedure-related deaths were observed in any of the patients. The mean total procedural time was 255.2 ± 48.4 minutes, the mean fluoroscopy time was 28.6 ± 8.2, and the mean ablation time was 29.6 ± 8.7 minutes (Table 2).

**Table 2:** Procedural characteristics of the study population.

Epicardial mapping (n,%)	20 (100)
Epicardial ablation (n,%)	19 (95.0)
Endocardial mapping (n,%)	11 (55.0)
Endocardial ablation (n,%)	7 (35.0)
VT induction during the procedure (n,%)	11 (55.0)
Substrate based ablation (n,%)	9 (45.0)
Retrograde transaortic approach (n,%)	7 (35.0)
Transseptal approach (n,%)	2 (10.0)
VT localization*	
LV Apex	8 (40.0)
LV Basal lateral region	3 (15.0)
LV Periaortic region	3 (15.0)
LV Basal septum	2 (10.0)
Epicardial LV posterobazal region	1 (5.0)
Epicardial RV bazal free wall	6 (30.0)
Epicardial RVOT	2 (10.0)
Endocardial RV bazal free wall	3 (15.0)
Supportive treatments during the procedure	
Norepinephrine infusion (n,%)	7 (35.0)
Dobutamine infusion (n,%)	8 (40.0)
Dopamine infusion (n,%)	2 (10.0)
CPR during the procedure (n,%)	1 (5.0)
Total procedural time (min)	255.2 ± 48.4
Fluoroscopy time (min)	28.6 ± 8.2
Ablation time (min)	29.6 ± 8.7

Categorical variables are presented as number (percentage), and continuous variables are expressed as mean ± standard deviation.

CPR = cardiopulmonary resuscitation, LV = left ventricle, RV = right ventricle, RVOT = right ventricular outflow tract, VT = ventricular tachycardia.

* Some patients had more than 1 location of VT.

Patients were followed up for an average of 591.5 ± 385.3 days after the procedure. One patient developed a transient ischemic attack after ablation, and 4 patients died during follow-up, with 3 deaths attributed to end-stage heart failure and 1 to a VT storm (Table 3). All patients in our study had devices with defibrillator functionality. Specifically, 16 patients were implanted with an ICD, while 4 patients received a CRT-ICD. Thus, there were no patients without defibrillator support in the cohort. Among the 4 observed mortalities, all were attributed to progressive heart failure rather than arrhythmic events.

**Table 3:** Follow-up data of the patients.

Follow-up duration (days)	591.5 ± 385.3
Prodecure-related death (n,%)	0 (0)
TIA after the procedure (n,%)	1 (5.0)
Exitus during the follow-up	4 (20.0)
Exitus causes	
End-stage heart failure (n,%)	3 (15.0)
VT Storm (n,%)	1 (5.0)
Decompensated HF during the follow-up (n,%)	4 (20.0)
Acute renal failure during the follow-up (n,%)	2 (10.0)
VT recurrence during the follow-up (n,%)	5 (25.0)
Approach to VT recurrence	
Medical follow-up	3 (15.0)
Re-ablation	2 (10.0)

Categorical variables are presented as number (percentage), and continuous variables are expressed as mean ± standard deviation.

HF = heart failure, TIA = transient ischemic attack, VT = ventricular tachycardia.

## 5. Discussion

This study is significant as it presents data on the characteristics of patients undergoing percutaneous subxiphoid epicardial VT ablation, the preoperative evaluation of patients with comorbidities, anesthetic agent selection by anesthesiologists, monitoring of intraoperative complications, and ensuring patient safety for successful VT ablation procedures. GA is typically preferred in patients undergoing epicardial ablation for sustained VT. Various studies have examined the effects of sedation versus GA on VT inducibility and procedural success.^[12]^ In addition to providing better catheter stability by controlling respiratory movements, GA improves patient comfort. However, as previously mentioned, there are concerns that GA may suppress sympathetic activity, potentially complicating the induction of arrhythmias. Despite these concerns, no significant difference was found between the sedation and GA groups in terms of epicardial access rates and procedural success for VT.^[13]^ All patients included in the current study received GA. In the preprocedural period, patients were evaluated collaboratively by anesthesiologists and cardiologists, taking into account comorbidities, VT etiology, NYHA classification, LVEF percentages, PAINESD scores, pulmonary artery pressure values, and the presence of LV thrombus.

The PAINESD score is used to predict the risk of periprocedural acute hemodynamic decompensation in patients undergoing VT ablation procedures and consists of 8 risk factors: chronic obstructive pulmonary disease, age >60 years, use of GA, ischemic cardiomyopathy, NYHA class III or IV, ejection fraction <25%, VT storm, and diabetes mellitus. Although GA is a risk factor for acute hemodynamic compensation, it remains the primary anesthetic type for VT ablation, although the 2019 Expert Consensus Statement on Catheter Ablation of Ventricular Arrhythmias suggests avoiding it in idiopathic VT cases.^[14,15]^ A PAAIN-ESD score of 17 or greater, where GA contributes 4 points, has been associated with an elevated risk for adverse procedural outcomes, including acute hemodynamic decompensation.^[16]^ In this study, the mean PAINESD score was 16.2 ± 9.3, and the mean LEVF was 22.5% (16.5–43.0%). Of the patients, 80% had an implanted cardioverter defibrillator and 70% were classified as NYHA III or IV.

During the procedure, norepinephrine, dobutamine, and dopamine were administered to 7, 8, and 2 patients, respectively. The mean total procedural time was 255.2 ± 48.4 minutes. GA remains the best option for new techniques and for prolonged interventional procedures. In this study, anesthetic induction was tailored based on each patient’s low LVEF, and the propofol dosage was minimized by initially administering midazolam and fentanyl, considering kidney function. Rocuronium was used to facilitate intubation, with dosages adjusted according to patient comorbidities. Sevoflurane was used for maintenance. Anesthetic agents can alter ventricular depolarization and action potential directly via effects on ion channels and gap junctions or indirectly via autonomic nervous system modulation.^[17]^

During EP tests and ablation procedures, propofol is commonly used for sedation or GA, with little impact on the conduction system.^[18]^ Propofol does not appear to increase the duration of the action potential or QT interval when compared with volatile anesthetics.^[19]^ However, in patients with clinical VT or VT storm unresponsive to antiarrhythmia treatment, propofol is frequently used to blunt sympathetic output and help terminate or control VT.^[20,21]^ Propofol was primarily used for induction and maintenance due to its predictable pharmacokinetic properties and established safety in our practice. Since induction of VT could compromise hemodynamic stability, a substrate ablation approach was preferred. In unstable patients, ketamine was considered as an alternative.

Etomidate is often used for anesthesia in patients with significant cardiac dysfunction, although evidence of its role in VT ablation is limited.^[22]^ Etomidate could have been considered as an induction agent; however, it was not available in our hospital during the study period. Therefore, propofol and/or ketamine were administered according to hemodynamic status and anesthesiologist preference. Remimazolam, a new anesthetic with minimal cardiac effects, has been suggested as a safe option for patients with low LVEF and severe mitral regurgitation; however, it is not yet available in Türkiye.^[23,24]^ It is more effective than etomidate in maintaining hemodynamic stability, with fewer side effects in elderly hypertensive patients.^[25]^ Benzodiazepines, such as remimazolam, show little impact on EP evaluations.^[26]^ Volatile anesthetics, including sevoflurane, isoflurane, and desflurane, help reduce ventricular arrhythmias, and sevoflurane is commonly used in EP labs.^[27]^ Although sufentanil may prolong the QT interval, fentanyl and remifentanil have minimal effects, with fentanyl used for induction to maintain stability and prevent titration issues.^[28]^

Since its inception, significant advancements have been made in VT ablation. The introduction of electroanatomic mapping strategies such as substrate and pace mapping has transformed VT ablation procedures by reducing the need for intraprocedural VT induction.^[29]^ In the current study, VT induction during the procedure occurred in 55% of cases, with 1 patient requiring cardiopulmonary resuscitation, which was successfully completed. VT substrate-based ablation was performed in 45% of the patients.

The current I-VT score confirms that LVEF, age, electrical storm, cardiomyopathy type, and diabetes mellitus, which are all components of the PAINESD score, are important predictors of poor outcomes. Compared to the PAINESD score, the I-VT score provides a longer-term (one-year) estimation and more accurate prediction of mortality.^[30]^ VT ablation, a strategy for periprocedural anesthesia to achieve hemodynamic stability, is essential to maximize safety and effectiveness. Pre-procedure risk stratification can help optimize peri- and post-procedural care. A high PAINESD score is not an absolute contraindication to GA. Periprocedural acute hemodynamic decompensation can be triggered by hypotension due to recurrent VT/VF, the use of anesthesia, and cardiac stunning due to repeated ICD shocks. Periprocedural acute hemodynamic decompensation has disastrous repercussions and significantly increases the likelihood of post-procedural death.^[30,31]^ In several clinical settings, percutaneous LV assist devices have been demonstrated to effectively support hemodynamics in patients with acute heart failure.^[32]^ In this study, it was determined that none of the patients required mechanical circulatory support. Hybrid EP laboratories represent a recent advancement in medical facilities equipped with positive ventilation, sterilization, and surgical instruments. These laboratories aim to decrease the mortality and morbidity associated with procedural complications, while enhancing patient outcomes.^[33–35]^ Our hospital does not have a hybrid laboratory, but there is a surgical team in the back up.

In our clinic, we integrated the I-VT score with the PAINESD score for use in clinical practice to enhance risk assessment.

The strategy we used in our study for patients with high PAINESD and IVT risk scores was to closely monitor hemodynamic results and findings of congestion in the critical care unit both before and after the procedure. Noradrenaline was administered if the systolic blood pressure was <100 mm Hg. Additionally, the patients were monitored for hypoxemia using arterial blood gas and daily posterior anterior chest radiography, and if congestion was present, additional diuretics were administered.

This study has several limitations: The primary weakness of this study is its retrospective nature. The documentation, although initially designed for research purposes, may have certain intraprocedural elements that are underreported or inconsistently documented. More consistent and thorough data collection, especially with regard to intraprocedural occurrences, would be possible with a prospective approach. However, the infrequency of the technique and the requirement for a larger number of samples rendered a retrospective analysis more practicable.

It was a single-center, non-randomized study with a limited number of patients and the retrospective nature of the study. In addition, we did not compare the anesthetic protocol with other widely employed protocols for epicardial catheter ablation. Despite these drawbacks, we present our anesthetic management and relevant follow-up data for a very high-risk patient population.

## 6. Conclusion

Stabilization of the patient, pain control, appropriate depth of anesthesia, and prevention of complications are critical for the success of anesthetic management strategies in patients undergoing percutaneous subxiphoid epicardial ablation for sustained VT. Therefore, the anesthesia team should develop a plan tailored to the unique needs of each patient. Although GA is generally preferred for epicardial VT ablation, an individualized approach is essential to ensure patient safety and achieve successful procedural outcomes.

## Author contributions

**Conceptualization:** Ferda Yaman, Tuba Akyuz, Ezgi Çamli Babayigit, Taner Ulus.

**Data curation:** Tuba Akyuz, Ezgi Çamli Babayigit.

**Formal analysis:** Ezgi Çamli Babayigit.

**Investigation:** Ferda Yaman, Tuba Akyuz, Ezgi Çamli Babayigit, Taner Ulus.

**Methodology:** Ferda Yaman, Ezgi Çamli Babayigit, Taner Ulus.

**Writing – original draft:** Ferda Yaman.

**Writing – review & editing:** Taner Ulus.
